# Electrokinetic Mixing for Improving the Kinetics of an HbA1c Immunoassay

**DOI:** 10.1038/s41598-019-56205-4

**Published:** 2019-12-27

**Authors:** Emir Yasun, Travis Trusty, Rania W. Abolhosn, Nigel J. Clarke, Igor Mezić

**Affiliations:** 1 Department of Mechanical Engineering and Biological Nanostructures Laboratory, California NanoSystems Institute (CNSI), University of California, Santa Barbara, Santa Barbara, CA 93106 USA; 2 iFluidics, Integrated Fluidics, 75 Robin Hill Rd, Goleta, CA 93117 USA; 3 Quest Diagnostics Nichols Institute, Advanced Technology R&D, 33608 Ortega Hwy, San Juan Capistrano, CA 92675 USA

**Keywords:** Biochemical assays, Assay systems, Diabetes

## Abstract

The efficiency of the diagnostic platforms utilizing ELISA technique or immunoassays depends highly on incubation times of the recognition elements or signaling molecules and volume of the patient samples. In conventional immunoassays, long incubation times and excess amounts of the recognition and signaling molecules are used. The technology proposed here uses electrokinetic mixing of the reagents involved in a sandwich immunoassay based diagnostic assay in electrode-enabled microwell plates in such a way that the incubation times and volumes can be reduced substantially. The integration of the electrodes at the bottom of the conventional microwell plates ensures that the motions of the liquid flows in the wells can be controlled through the application of high frequency AC current along these electrodes. The strategy to generate chaotic mixing by modification of standard multiwell plates, enables its use in high throughput screening, in contrast to microfluidic channel-based technologies that are difficult to incorporate into conventional plates. An immunoassay for detection of glycated hemoglobin (HbA1c) that can reveal a patient’s average level of blood sugar from the past 2–3 months instead of just measuring the current levels and thereby constitutes a reliable diabetes monitoring platform was chosen as a pilot assay for technology demonstration. The overall incubation time for the assay was reduced by approximately a factor of five when electrokinetic mixing was employed. Furthermore, when the quantity of the reagents was reduced by half, almost no distinguishable signals could be obtained with conventional immunoassay, while electrokinetic mixing still facilitated acquisition of signals while varying concentration of the glycated hemoglobin. There was also a substantial difference in the signal intensities especially for the low concentrations of the HbA1c obtained from electrokinetic mixing assisted and conventional immunoassay when the quantity of the reagents and incubation times were kept constant, which is also an indication of the increase in bioassay efficiency. The electrokinetic mixing technique has the potential to improve the efficiency of immunoassay based diagnostic platforms with reduced assay time and reagent amounts, leading to higher throughput analysis of clinical samples. It may also open new avenues in point of care diagnostic devices, where kinetics and sampling size/volume play a critical role.

## Introduction

Rapid, accurate and affordable quantitation of disease markers is crucial for development of efficient diagnostic tests and biosensors^1,2^. Early diagnosis can facilitate therapeutic intervention and increase the chance of recovery and survival especially for diseases like cancer^3–6^. It can also help decrease the chance of transmission of communicable diseases like STIs^7,8^ (sexually transferred infections). Besides the need for development of affinity probes specific and selective towards their target molecule, there is also a need for increase in efficiency of the current diagnostic platforms. In ELISA (enzyme-linked immunosorbent assay) or other immunoassay based diagnostic platforms, molecular recognition elements such as antibodies, proteins, and oligonucleotides are usually used in excess amounts, and they are incubated with their corresponding targets for a significant time to ensure the reactions go to completion with all the possible binding sites saturated. This affects the timing and cost of the assays adversely, and has a negative impact on the sample throughput for clinical screening. In addition, the high cost of the test components may prevent testing frequent enough to detect serious changes in the disease state. In this work, we propose the use of electrokinetic mixing to improve the kinetics and reagent utilization of biological assays.

One of the main technical challenges to accelerate biochemical reactions is mixing. Over the last 20 years many micro-mixers have been investigated. Mixers are intended to increase the contact area between two fluids (or particles) by diffusion, lamination (e.g. superimposition of flow layer) or successive folding and stretching of flows leading to what is called chaotic advection. Chaotic advection achieved in 3D flows or time dependent 2D flows increases mixing exponentially as opposed to diffusion or lamination based mixing which rather follow an algebraic mixing rate (*e.g*. t^−1^)^9^. From a technological point of view, mixers can be divided into 2 categories: passive and active. Passive mixing is due to the configuration of the channels (e.g. grooves^10^, serpentine channels^11^), whereas active mixing consists of applying external forces to the fluid (e.g. pressure control, electrokinetics, magneto hydrodynamics).

In 2000, Liu *et al*. designed 3D serpentine channels to stir the fluid in 3 dimensions at channel bends. However this passive mixer was complicated to fabricate and had poor efficiency for low Reynolds Number flows, since the vortices generated were only due to fluid inertia^11^. For this reason, the device has been enhanced adding lamination flows to chaotic advection^12^. The influence of the channel geometry has been then studied and optimized^13^. Stroock *et al*. designed non-symmetric ridges along the micro-channel so as to generate alternating bigger and smaller vortices generating thin superimposed layers of fluid^10^. This concept was similar to time dependent overlapping vortices suggested by Aref^14^, with a 3D steady flows instead.

Chaotic active micro-mixing at the intersection of micro-channels has been achieved with perturbation flows controlled by pressure controller^9,15^. In the wake of the work of Solomon and Mezic^16^ the importance of spatiotemporal resonance frequency has been studied in these mixers^17^. However, for those active and passive mixers, the system requires pressure controllers or syringe pumps, which cannot be easily integrated into a lab on a chip or microtiter plate setting.

In that regard, electrokinetic microfluidic actuators have been developed in lab on chip platforms for active mixing^18,19^. Stirring in high conductive buffers has mostly used steady electrothermal and to a lesser extent, buoyancy vortices. As for electroosmotic micro-mixers, used in low conductive environments, have been mostly designed to reproduce flow topology of previous mixers such as Stroock’s passive mixer^20–22^ or even cross channel active mixers^23^. Most of these cross-channel mixers turned out to be less efficient than the ones controlled with a pressure-based actuation, since electrosmotic pumps are not powerful enough to cut off the flow of the main channel. This issue has been partially treated by Lin *et al*. who increased the perturbation flow by pulling back the fluid into the opposite side channel^24^. Coleman *et al*. enhanced the mixing without any chaotic advection but by locally narrowing the channel so as to locally decrease the Péclet number thereby accelerating diffusive mixing^25^. Yan *et al*., coupled electrosmotic pulsation with chaotic advection-based passive mixing using patterned block channel geometry^26^. Others have been using electrokinetic instability to perform chaotic mixing^27–29^. This instability, which results from a competition between the electrically viscous force and the diffusion and the dragging force, occurs at the interface between two fluids of different conductivities.

Microwell plate-based assays typically utilize active mixing regimes induced by external stimuli such as orbital shaking/vortexing, liquid handler pipetting, magnetic stirring, and acoustics to maintain the mixing^30^. The dependence on external stimuli prevents detailed mixing control. Elecrokinetic mixing has so far been mainly used in microchannels. To the best of our knowledge, ours is the first platform to enable mixing actuation embedded into the microtiter wells or multiwell plates. This provides both conformation to the standard shape factors used in industry, as well as flexibility in design of mixing protocols. In this paper we use two such protocols, that we name symmetric (double gyre) and dynamic (time-dependent double gyre) mixing regime. The symmetric regime is the one in which the flow in the well features two vortices (gyres). The mixing proceeds largely by shearing action combined with rotation. The dynamic regime induces chaotic advection by overlapping the domain of two vortices in a time-periodic protocol. The symmetric regime is expected to produce mixing that uniformizes the concentration algebraically (as 1/t) in time, while dynamic mixing regime is expected to do so exponentially (e^−lt^)^31^. On the other hand, besides the microfluidic devices and multiwell plates paper-based lab on a chip designs are recently popular as well^32–34^. Despite being a cost-efficient platform for mixing or separation purposes, paper-based bioassays are not as commonly used as the multiwell plates for high throughput screening since the detection techniques (i.e. colorimetric, rather qualitative) used are not as sensitive as the ones used for multiwell plates (i.e. fluorescence) and reproducible mass production depends on a lot of parameters so most of the immunoassays are designed and compatible for multiwell plate platforms.

The electrokinetic flow mode used for mixing of high conductive solutions in microchannels is AC electrothermal (ACET). In this mode, reducing the AC voltages and increasing the frequencies enable circumvention of the electrolytic reactions occurring around the electrode surface. This requirement leads the optimization of the applied voltage and frequency (e.g. 3.5 V (7 Vpp) with a 1 MHz frequency (instead of using kV or Hz) was used here, see the results section). Another force contributing to the overall mixing in our work is buoyancy, leading to buoyancy induced flow (BF). ACET flows are due to the conductivity and permittivity gradients of the solution, whereas BF results from the density gradients. Both gradients are induced by Joule heating resulting from the application of voltages to the electrodes that are in contact with the highly conductive solution. This non-uniform heating resulted from non-uniform electric field causes the change in conductivity and permittivity of the solution. These gradients are responsible for the ACET-and BF induced vortical motion inside the solutions. ACET and BF induced vortical motions happen at the lower (<1000 µm) and higher (>1000 µm) subregions of the solution, respectively.

We induce electrokinetic mixing in custom made microwell plates assembled with a PCB (printed circuit board) at the bottom (Supporting Information, S1). The PCBs were patterned with three gold electrodes for each row of the wells (the bottom of each well is in contact with the gold electrodes) arranged in parallel. They are driven by a signal generator that generates AC square wave voltage. The desired square wave voltages and their frequencies can be adjusted to change the speed of the flow. The phase changes on the electrodes can manipulate the direction of the flow induced in the solutions placed inside the wells. The appropriate switch in the phase of the voltage induced on the electrodes can lead to the chaotic mixing^14^ in the dynamic regime. This type of mixing induced by chaotic advection facilitates contact of the molecules participating in the reaction and improves the diffusion limited kinetics. To the best of our knowledge, this is the first mixing platform to embed mixing actuation ability directly into the microwell plates, as opposed to using external actuators to mix.

In this work, as a consequence of applying electrokinetic mixing, we observed a dramatic decrease in the incubation times (up to 5 times) and amounts of reagents used (the reduction of the amount of the reagents by half didn’t affect the results). Additionally, we obtained huge improvements in fluorescence intensities, especially at the low concentrations of the analyte (up to 39 times) when electrokinetic mixing was applied. Thus, this method is capable of increasing the efficiency of diagnostics assays in terms of speed and cost.

A sandwich immunoassay was tested as a pilot assay to demonstrate the effect of electrokinetic mixing for the improvement of reaction kinetics. The assay detects glycated hemoglobin (HbA1c) to determine a surrogate marker for the patient’s control of their blood sugar over the past 2–3 months. When glucose levels are elevated in blood, they react with hemoglobin, an oxygen carrier protein present in red blood cells and form the glycated hemoglobin. This is a non-enzyme mediated chemical reaction. The assay measures this glycated hemoglobin to find the percentage of glycated hemoglobin present in the sample^35,36^. Since the life span of the red blood cells are about 3–4 months, that is why the immunoassay is capable of imputing the level of diabetic control over the past 3 months within the patient^36^. In contrast to other diagnostic kits capable of revealing the current/daily glucose levels in blood, HbA1c is an important blood test to diagnose type 2 diabetes and monitor the progression of diabetes over a period of months^36^. Thus, measuring HbA1c levels provides a more reliable and long term results for the patient’s diabetic control and holds promise for improvements leading to better compliance with dietary and lifestyle modifications prescribed and reducing the risk of diabetes related complications^37^.

The immunoassay was employed to detect HbA1c in buffer solutions. The kinetics results obtained by conventional and electrokinetic mixing assisted assay were compared to establish the efficacy and applicability of electrokinetic mixing for diagnostic and biological assays.

## Materials and Methods

### Materials

HbA1c Capture Antibody (cat H01292M) and HbA1c Detect Antibody (cat H01291M) were purchased from Meridian Life Science. Human Hemoglobin A1c full length protein (cat ab82273) was obtained through Abcam. Pierce Streptavidin Magnetic Beads (cat 88816) and EZ-Link Sulfo-NHS-SS-Biotin (cat 21331; used to modify the HbA1c Capture Antibody) were bought from ThermoFisher Scientific. Alexa Fluor 647 Monoclonal Antibody Labeling Kit (cat A20186; used to modify the HbA1c Detect Antibody) was ordered from Invitrogen. 10x PBST (with 0.5% Tween 20, pH = 7.4) (cat J63596) from Alfa Aesar was used throughout these experiments.

### Conjugation of the antibodies to the magnetic beads

The magnetic beads (MBs, 10 mg/mL) were first washed utilizing a magnetic particle separator stand and PBST (0.05% Tween 20, 1x Phosphate-buffered saline, pH 7.4) for 3 times. Then 2 mg of beads were incubated with 110 µg of capture IgG for HbA1c in 300 µL PBST at room temperature for 1 hour while shaking. Following the incubation step, MBs were washed with PBST twice. The final concentrations of MBs and capture IgG were arranged to be 2 mg/mL and 0.11 µg/µL in PBST, respectively.

### Preparing the standard solutions of HbA1c

1000, 100, 10, 1, 0.1, 0.01, 0.001 µg/mL standard solutions of HbA1c were prepared in PBST from the stock solution of HbA1c.

### Step 1 (capture) of HbA1c immunoassay

20 µL of capture antibody-conjugated magnetic beads (2 mg/mL MBs) were incubated with 10 µL of the standard HbA1c for 1 hour at room temperature in microwell plates. Following the incubation, the wells were placed on a magnetic bed and washed for 3 times with 35 µL of PBST while aspirating them. (A custom-made aspirator was set up that was connected to the vacuum).

### Step 2 (detection) of HbA1c immunoassay

After washing, 20 µL of 50 nM of the detection antibody previously modified with Alexa Fluor was incubated with the magnetic beads-HbA1c complex at room temperature for 30 minutes. After the incubation, the wells were placed on a magnetic bed and washed/aspirated twice with PBST.

### Quantitation of HbA1c

Finally, after washing, the “detection antibody-HbA1c-magnetic beads” complex was dispersed in 100 µL PBST in each well and the multiwell plate was placed in Tecan M200 multiwell plate reader. The fluorescence intensities for the wells were measured at the emission wavelength of 673 nm and the excitation wavelength of 640 nm.

### Electrokinetic mixing method

Electrokinetic mixing is performed through the induction of AC electrothermal and buoyancy-driven flows. Buoyancy arises due to electrical current-induced localized temperature and density difference; thus, we label the whole flow electrokinetic. These flows are formed by placing three parallel electrodes in contact with a conductive solution as described by Loire *et al*.^38^. By varying the voltage potential on each individual electrode, one can form vortices within the well containing the solution. The character of particle/analyte motions within the well are determined by the strength and directionality of the controlled electric field, as well as by buoyancy effects caused by temperature and density differences. In these experiments, we make use of two mixing regimes: symmetric mixing and dynamic mixing. Symmetric mixing is induced by a sequence of positive, negative and positive phases of the first, second (middle) and third electrodes, respectively [+, −, +]. This configuration will form two symmetric vortices (a double gyre) on the left and right sides of the well. Dynamic mixing automatically cycles through the following phases: [0, −, +] for 15 sec, fully off for 0.5 sec, then [+, −, 0] for 15 sec This generates the chaotic mixing, as explained further in the results and discussion section.

All experiments were done using a 1 MHz AC square wave supplied to each electrode (unless grounded). A voltage of 3.5 V (7 V peak to peak) was chosen based on visual inspection of micro fluorescent bead motion in PBST at various voltages. These videos are available in the supporting information (Supporting Information, Videos 2). While a higher voltage may allow for better mixing, one can observe particle aggregation and bubble formation (which can be an indication of an electrolytic reaction, electrolysis) at the voltages of 4 V and above. The conventional HbA1c immunoassay used in this experiment require the suggested standard incubation times of 60 minutes (step 1) and 30 minutes (step 2). In electrokinetic mixing assisted assays, either 5 or 10 minutes of incubation times were chosen for the incubations in Step 1 and Step 2 of the HbA1c immunoassay.

## Results and Discussion

**Figure 1:**
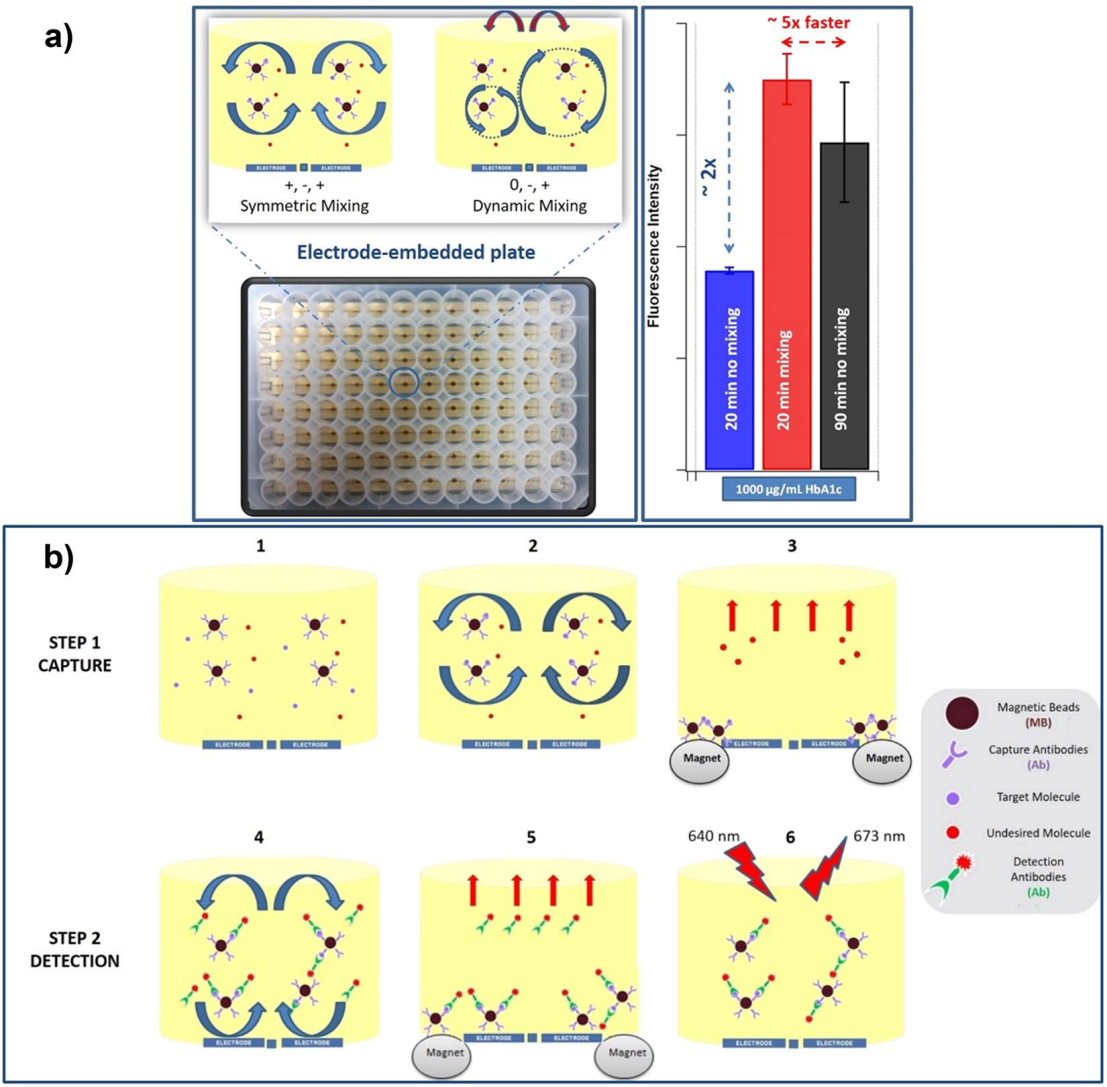
(**a**) Different electrokinetic mixing regimes that take place in the wells of an electrode-enabled microtiter plate. Different mixing regimes can be controlled by changing the phases of the electrodes (+, − and 0). The blue arrows show the direction of the motion and the red arrows show the exchange of the different size vortices every 15 seconds between the two sides of the wells induced by the switch of the electrode phases from [0 − +] to [+ − 0]. (**b**) The overall scheme of the electrokinetic mixing assisted HbA1c sandwich assay. Each yellow receptacle represents inside of a well and the blue bars at the bottom of the wells represent the gold electrodes. Step 1 (1, 2, 3) and Step 2 (4, 5, 6) is for capture and detection of HbA1c, respectively (1). Introduction of the capture antibody-conjugated magnetic beads and HbA1c (2), Electrokinetic mixing of the capture antibody-conjugated magnetic beads and HbA1c (3), Aspirating/washing step for the excess reagents after the incubation of the capture antibody-conjugated magnetic beads and HbA1c (4), Introduction and electrokinetic mixing of the Alexa Fluor-modified detection antibody and “magnetic bead-HbA1c” complex (5), Aspirating/washing step of the excess detection antibodies (6), Taking the emission spectrum of the assay to measure the extent of the captured HbA1c (Excitation: 640 nm and Emission: 673 nm).

In this work, a two-step assay detecting HbA1c was adopted to compare the results obtained with conventional immunoassay, where no mixing was applied, and the electrokinetic mixing assisted assay. This sandwich assay consists of capture and detection steps. For the conventional assay (no mixing), in step 1, glycated hemoglobin was incubated with the capture antibody-conjugated magnetic beads (MBs). Following the aspirating/washing steps utilizing a magnetic bed to remove the excess reagents, the magnetic bead-HbA1c complex was incubated with detection antibody previously modified with Alexa Fluor 647. After the last aspirating/washing steps, the fluorescence spectra of the samples were taken to measure the captured HbA1c (Scheme 1). For the electrokinetic mixing assisted assay, in each step, the incubations were assisted by electrokinetic mixing for either 5 or 10 minutes as it can be seen in the overall scheme of the diabetes sandwich assay in Fig. 1b and Video 1 (Supporting Information, Video 1). The electrokinetic mixing mode could also be changed by varying the phases of the electrodes to be either symmetric or dynamic mixing (Fig. 1a).

**Figure 2:**
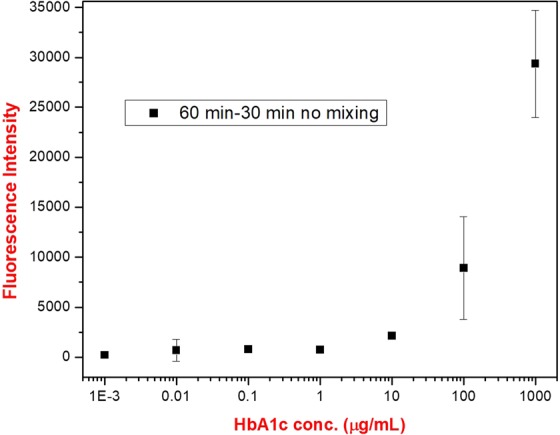
The conventional HbA1c immunoassay results after step 1 (capture, 60 min) and step 2 (detection, 30 min) of the assay. The times in the legend of the graph represent the step 1 and step 2 incubation times, respectively. The fluorescence intensities were acquired at emission and excitation wavelengths of 673 nm and 640 nm in a multiwell plate reader, respectively.

The HbA1c immunoassay was first tested using the standard incubation times, where no electrokinetic mixing was utilized. Varying concentrations of HbA1c in buffer solution were tested. The incubation times were 60 minutes and 30 minutes for step 1 (capture) and step 2 (detection), respectively. As can be seen in Fig. 2, quantifiable signals could be obtained for HbA1c concentrations of 10 µg/mL and above. A baseline was observed at the concentrations below 1 µg/mL. Thus, we focused the reaction kinetics comparisons between the conventional and electrokinetic mixing assisted assays on the HbA1c concentrations at 1 µg/mL and above.

**Figure 3:**
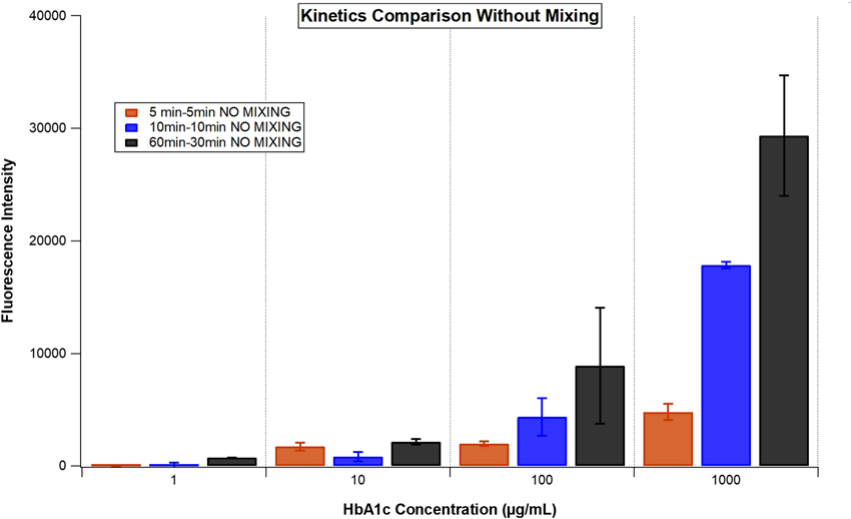
The fluorescence intensity comparison of the conventional HbA1c immunoassays with 5–5 minutes, 10–10 minutes, 60–30 minutes protocols where the numbers refer to the incubation times for step 1 - step 2 of the immunoassays. The fluorescence intensities were acquired at emission and excitation wavelengths of 673 nm and 640 nm, respectively, in a multiwell plate reader.

In order to ensure that the antibody-antigen interactions don’t go to completion (equilibrate) in shorter incubation times than the standard 60–30 minutes incubation protocol, we compared the fluorescence intensities of the conventional HbA1c immunoassays with the incubation times of 5–5 minutes, 10–10 minutes and 60–30 minutes for step 1-step 2 of the assay. As can be clearly seen in Fig. 3, at higher concentrations of HbA1c, the immunoassay that employing shorter incubation times for both steps couldn’t match the fluorescence intensities obtained for the immunoassay employing incubation times of 60 and 30 minutes for step 1 and step 2, respectively (Fig. 3). Thus, there is shown to be room for improvement in the kinetics of this immunoassay that could potentially be provided by the electrokinetic mixing method, especially for shorter incubation times.

**Figure 4:**
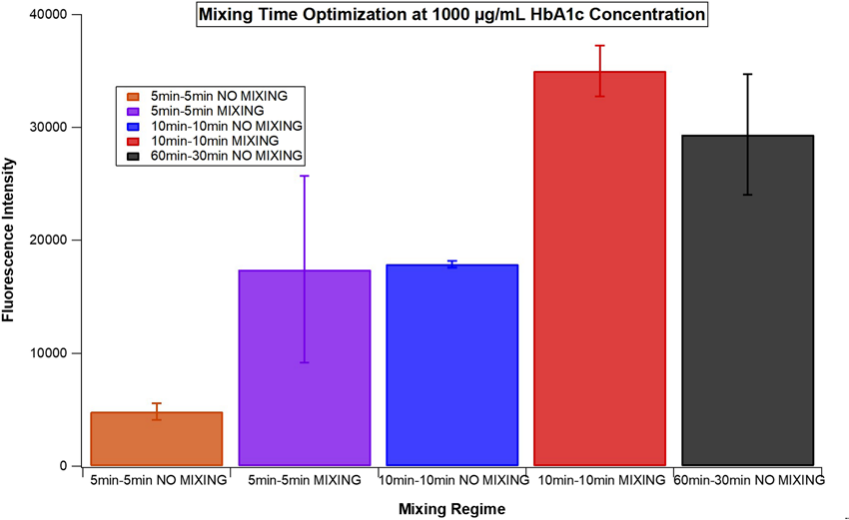
The fluorescence intensity comparison of the HbA1c conventional and electromixing assisted immunoassays with 5–5 minutes, 10–10 minutes (both assays) and 60–30 minutes (only conventional) incubation times for step 1 - step 2 of the assay. The fluorescence intensities were acquired at emission and excitation wavelengths of 673 nm and 640 nm in a multiwell plate reader, respectively.

In order to optimize the incubation time for the electrokinetic mixing application, the fluorescence intensities of the electrokinetic mixing assisted immunoassays with the incubation times of 5 or 10 minutes for each step were compared to the conventional immunoassay (where the standard incubation times, 60 and 30 minutes for step 1 and step 2 were employed) at the highest concentration of HbA1c (1000 µg/mL). When electrokinetic mixing was applied for either 5 or 10 minutes for each step of the assay, the fluorescence intensities obtained were higher than the ones obtained for the conventional assay with incubation times of 5 or 10 minutes for each step (Fig. 4). Moreover, the fluorescence intensities for the electrokinetic mixing assisted assay with the incubation times of 10 minutes for each step were higher than the ones obtained for the electrokinetic mixing assisted assay with the incubation times of 5 minutes for each step. Compared to the fluorescence intensities from the conventional assay (60–30 minutes), comparable and even higher fluorescence intensities were obtained by the electrokinetic mixing assisted assay with the incubation times of 10 minutes for each step (Fig. 4). Therefore, the duration of the electrokinetic mixing application was selected to be 10 minutes for each step.

**Figure 5:**
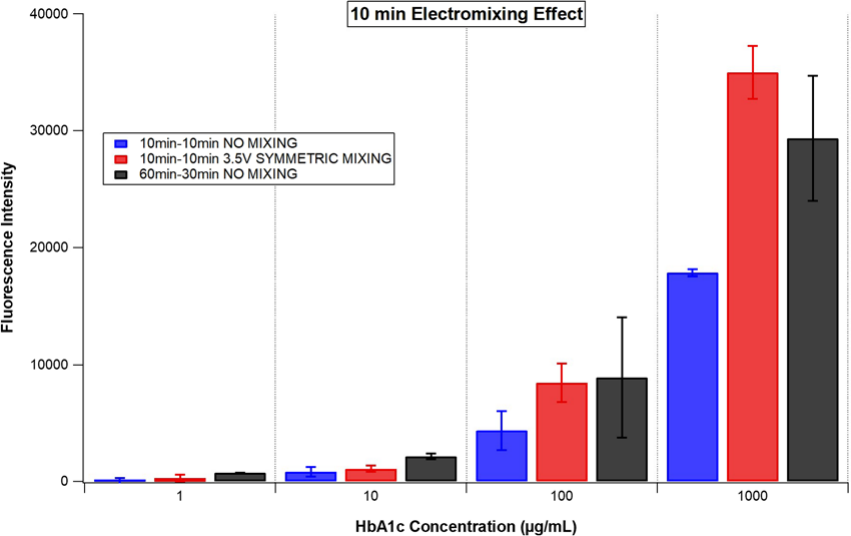
The fluorescence intensity comparison of the HbA1c conventional and electro-mixing assisted immunoassays while 10–10 minutes (both assays) and 60–30 minutes (only conventional) were used as incubation times for step 1-step 2 of the assay. The fluorescence intensities were acquired at emission and excitation wavelengths of 673 nm and 640 nm in a multiwell plate reader, respectively.

After electrokinetic mixing was applied for 10 minutes for each step of the assay, the fluorescence intensities obtained were all higher than the ones obtained for the conventional assay with incubation times of 10 minutes for each step (Fig. 5). In addition, fluorescence intensities obtained by the electrokinetic mixing assisted assay (only 10 minutes for each step) were comparable and even slightly higher compared to the ones obtained for the conventional assay with suggested incubation times (60–30 minutes) at higher concentrations of HbA1c (100 and 1000 µg/mL) (Fig. 5). This clearly indicates that electrokinetic mixing improves the reaction kinetics of this assay. Namely, in order to obtain similar fluorescence intensities, 20 minutes of overall incubation time was required for the electrokinetic mixing assisted assay, whereas the conventional assay required an overall incubation time of 90 minutes (Fig. 5). This suggests the overall incubation time of the HbA1c immunoassay could be reduced by approximately a factor of five (from 90 minutes to 20 minutes).

**Figure 6:**
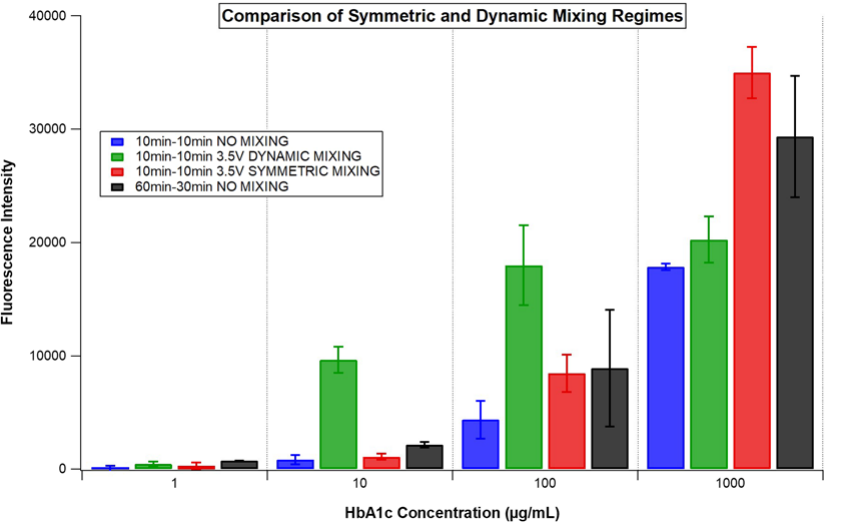
The comparison of the different electromixing regimes induced by the electrode phase changes with the same application times (10 and 10 minutes for step 1 and step 2 of the HbA1c immunoassay, respectively). The fluorescence intensities were acquired at emission and excitation wavelengths of 673 nm and 640 nm in a multiwell plate reader, respectively.

To improve fluorescence intensities obtained by short-time protocols at the lower concentrations of HbA1c (1 and 10 µg/mL, Fig. 5), a different electrokinetic mixing regime, known as dynamic mixing, was employed to ascertain the impact on the generated fluorescence intensities (Fig. 6). In contrast to symmetric mixing, dynamic mixing that induces asymmetry in vortex (gyre) sizes at two sides of the well allows the contents of the solution to travel rapidly to both sides of the well. The asymmetric double gyres exchange sides of the wells with the switch of the electrode’s phases. The asymmetry of the vortices and the dynamic exchange of the location of the vortices are responsible for the contents of the solution to across the well by inducing chaotic advection process inside the well. When dynamic mixing was applied for 10 minutes for each step of the HbA1c immunoassay, the fluorescence intensities obtained at the lower concentrations of HbA1c were even higher compared to the ones obtained for the conventional HbA1c immunoassay with standard incubation times (60–30 minutes, Fig. 6). However, the highest fluorescence intensities were obtained with the symmetric mixing at the highest concentration of HbA1c (1000 µg/mL) compared to the dynamic mixing assisted and conventional HbA1c immunoassay (Fig. 6). This is explained by the fact that, while in the symmetric mixing mode the vortices mix for all the available time, in the dynamic mixing protocol only half of the content of the well is mixed during any period. There is a pause period of the dynamic mixing when the electrode phases were switched. Dynamic mixing allows one half part of the well to mix in the mixing mode and then at the end of this mixing (when the switch occurs) it transfers some of those mixed reagents to the other half part of the well and then that part continues to mix and transfers some of the mixed reagents to other part of the well and so on. So it allows the switching of the molecules from one half of the well to the other part of the well. It is a different type of mixing mode, which is not continuous like symmetric mixing. In symmetric mixing both half parts of the wells were continuously mixed (without the reagent transfer between the half parts of the wells).

Dynamic mixing produced higher fluorescence intensities for the low concentrations of HbA1c since diffusion limitation is more influential in the overall reaction kinetics and can be overcome by the improved mixing of the analytes.

**Figure 7:**
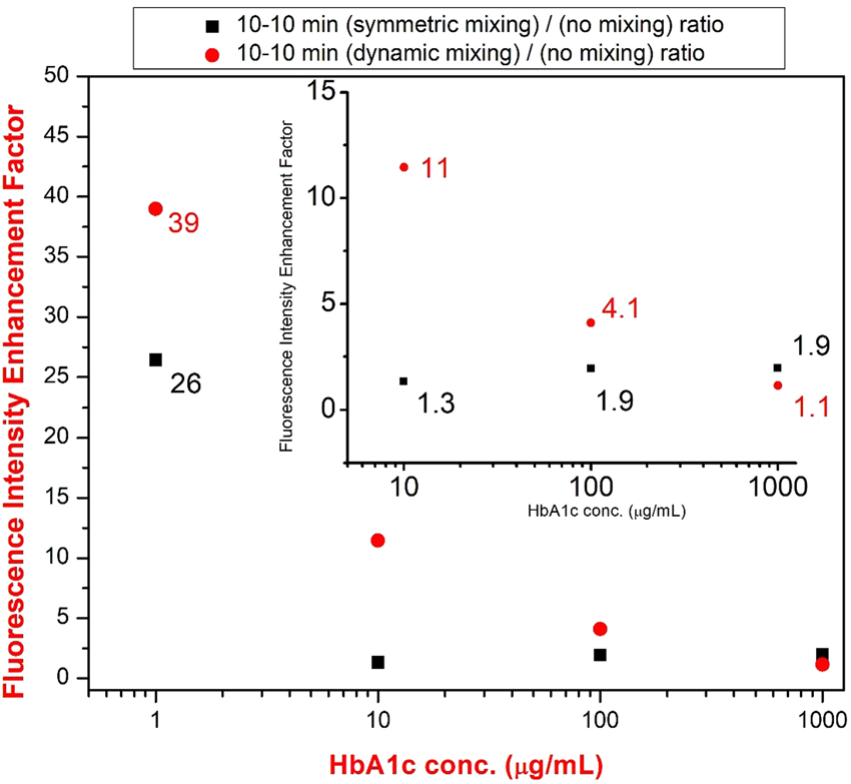
Fluorescence intensity enhancement factor comparison between the symmetric and dynamic mixing regimes. The ratios of the fluorescence intensities of the mixing assisted and conventional assay were compared for each concentration of the HbA1c.

When the fluorescence intensities were compared for the mixing and no mixing cases both symmetric and dynamic mixing regimes improved the fluorescence intensities of the assay with increasing concentrations of HbA1c. The improvement effect was more evident at lower concentrations of HbA1c (i.e. 39 and 26 folds for dynamic and symmetric mixing, respectively) and it decreases with the concentration increase of the HbA1c. This nonlinear decay in the improvement effect can be seen in Fig. 7. Thus, electrokinetic mixing has a higher impact in improving the kinetics of the lower concentrations of the analytes where diffusion limitation is higher. The reaction rate consists of components that depend on concentration of the reactants and the kinetics of the reaction itself. If the kinetics of the reaction are relatively fast, the rate will depend on the concentration only. Thus, at the low concentrations, the diffusion limitation is significant. In other words, while higher concentration will result in higher rates of reaction, lower concentration will result in lower rates of reaction in the solutions. Electrokinetic mixing helps improve concentration gradients and thus enhance the effective diffusivity, thus improving the reaction rate.

**Figure 8:**
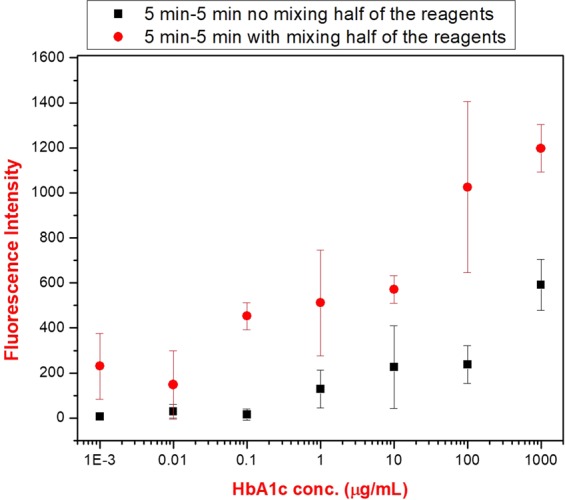
The comparison of the fluorescence intensities when half of the reagents were used for electrokinetic mixing assisted (symmetric mixing) and conventional HbA1c immunoassay with incubation times of 5–5 minutes for step 1-step 2 of the assay. The fluorescence intensities were acquired at emission and excitation wavelengths of 673 nm and 640 nm in a multiwell plate reader, respectively.

Electrokinetic mixing was also able to lower the volumes of each reagent used in the assay. Excess reagents are usually used to ensure the reactions equilibrate within the suggested incubation time, which is usually excessive as well. In order to demonstrate the applicability of electrokinetic mixing for removing the need for the use of excess reagents, half volume of the each reagent (capturing antibody-conjugated magnetic beads, HbA1c and the detection antibody) from the HbA1c immunoassay assay were used for both conventional and electrokinetic mixing assisted assays with the incubation times of 5–5 minutes for step 1- step 2 of the immunoassay (from 30 µL to 15 µL as a total volume). When the fluorescence intensities were compared for the electrokinetic mixing assisted and conventional assay, besides having higher fluorescence intensities compared to the ones obtained for the conventional assay, it can be seen from Fig. 8 that the concentrations of HbA1c are above the baseline starting at 0.1 µg/mL in electrokinetic mixing assisted assay. In contrast, the fluorescence intensities of the conventional assay were all close to the baseline except for the HbA1c concentration of 1000 µg/mL (Fig. 8). This also indicated that electrokinetic mixing method has the potential to allow an immunoassay to function with a reduced amount of reagents, which can lower the cost of running the assay substantially.

## Conclusions

In this study, we have demonstrated that electrokinetic mixing can improve the kinetics and reagent utilization of diffusion-limited immunoassay based diagnostic platforms or other biological assays. To prove this, an HbA1c immunoassay that detects glycated hemoglobin was chosen as a pilot assay and its overall incubation time was reduced by approximately 5 times from 90 minutes to 20 minutes by utilizing electrokinetic mixing. Further, electrokinetic mixing was shown to help avoid the need for excess molecular recognition reagents in this immunoassay. We also showed that when the quantity of the HbA1c assay reagents was reduced by half (i.e. from 30 µL total volume to 15 µL), almost no distinguishable signals could be obtained with the conventional immunoassay, whereas electrokinetic mixing facilitated acquisition of signals with above baseline intensities. This demonstrated that electrokinetic mixing could enable more cost-efficient biological assays. Moreover, in the experiments presented here, there was a substantial difference in the signal intensities between electrokinetic mixing-enabled and the standard assay, especially at the lower concentrations of antigen, when the quantity of the reagents and incubation times were kept constant. Thus, electrokinetic mixing could be helpful for the low abundant target biomolecule detections.

Our methodology works optimally for the assays that are diffusion limited and thus require higher incubation times like the assay in this study. In order to optimize the protocol for a particular assay, the voltages should be optimized first with the assay buffer by observing the microfluorescent bead motions, as explained in the methods section. Subsequently, an experimental matrix of mixing times and volumes should be applied to the assay to find parameters for which signal intensities when the assay is performed without mixing and with the suggested incubation times are similar.

In summary, this methodology has potential to improve the efficiency of the current diffusion limited point of care devices and diagnostic platforms utilizing microwell plates so that rapid, accurate, cost-efficient and high throughput analysis of clinical samples may be achieved.

## Supplementary information

0.5V_1MHz_SymmetricMixing

1V_1MHz_SymmetricMixing

1.5V_1MHz_SymmetricMixing

2V_1MHz_SymmetricMixing

2.5V_1MHz_SymmetricMixing

3V_1MHz_SymmetricMixing

3.5V_1MHz_SymmetricMixing

4V_1MHz_SymmetricMixing

Supporting Information-Video 1

Supporting Information
