# Supplementary material for: Electrokinetic Mixing for Improving the Kinetics of an HbA1c Immunoassay
Source: Sci Rep. 2019 Dec 27;9:19885. doi: 10.1038/s41598-019-56205-4 (PMC6934526; doi:10.1038/s41598-019-56205-4)
Supplement: Supplementary file 10 — Supporting Information [file 41598_2019_56205_MOESM10_ESM.docx]

**Supporting Information**

**Electrokinetic mixing for Improving the Kinetics of an HbA1c Immunoassay**

**Emir Yasun^1,2^, Travis Trusty^1,2^, Rania W. Abolhosn^3^, Nigel J. Clarke^3^ and Igor Mezic^1*^**

^1^Department of Mechanical Engineering and Biological Nanostructures Laboratory, California NanoSystems Institute (CNSI), University of California, Santa Barbara, Santa Barbara, CA 93106, USA

^2^iFluidics**,** Integrated Fluidics, 75 Robin Hill Rd, Goleta, CA 93117, USA

^3^Quest Diagnostics Nichols Institute, Advanced Technology R&D, 33608 Ortega Hwy, San Juan Capistrano, CA 92675, USA

[emiryasun@engineering.ucsb.edu](mailto:emiryasun@engineering.ucsb.edu)

[emiryasun@ucsb.edu](mailto:emiryasun@ucsb.edu)

[emiryasun@chem.ucsb.edu](mailto:emiryasun@chem.ucsb.edu)

mezic@ucsb.edu*

a.
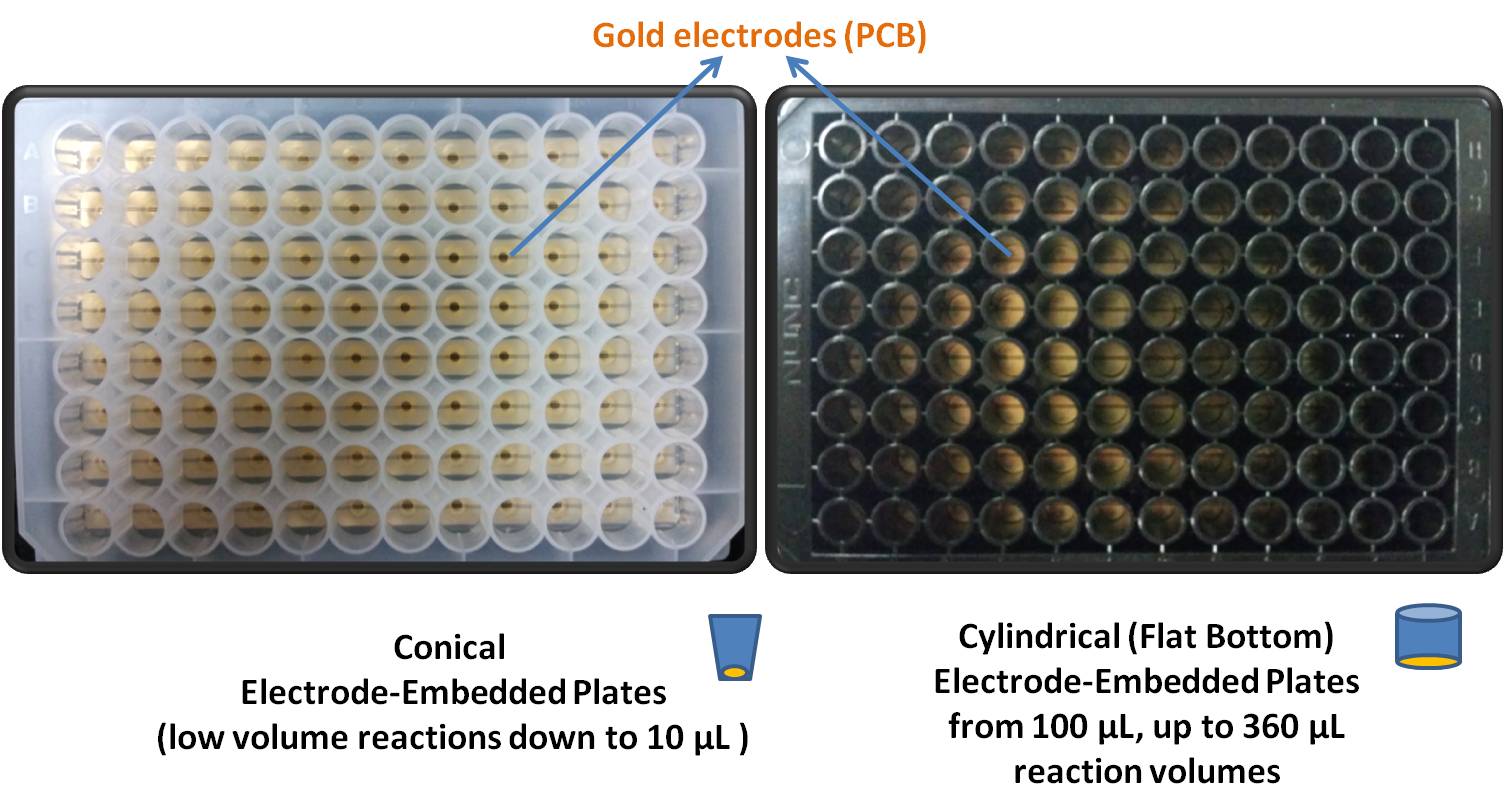


b.
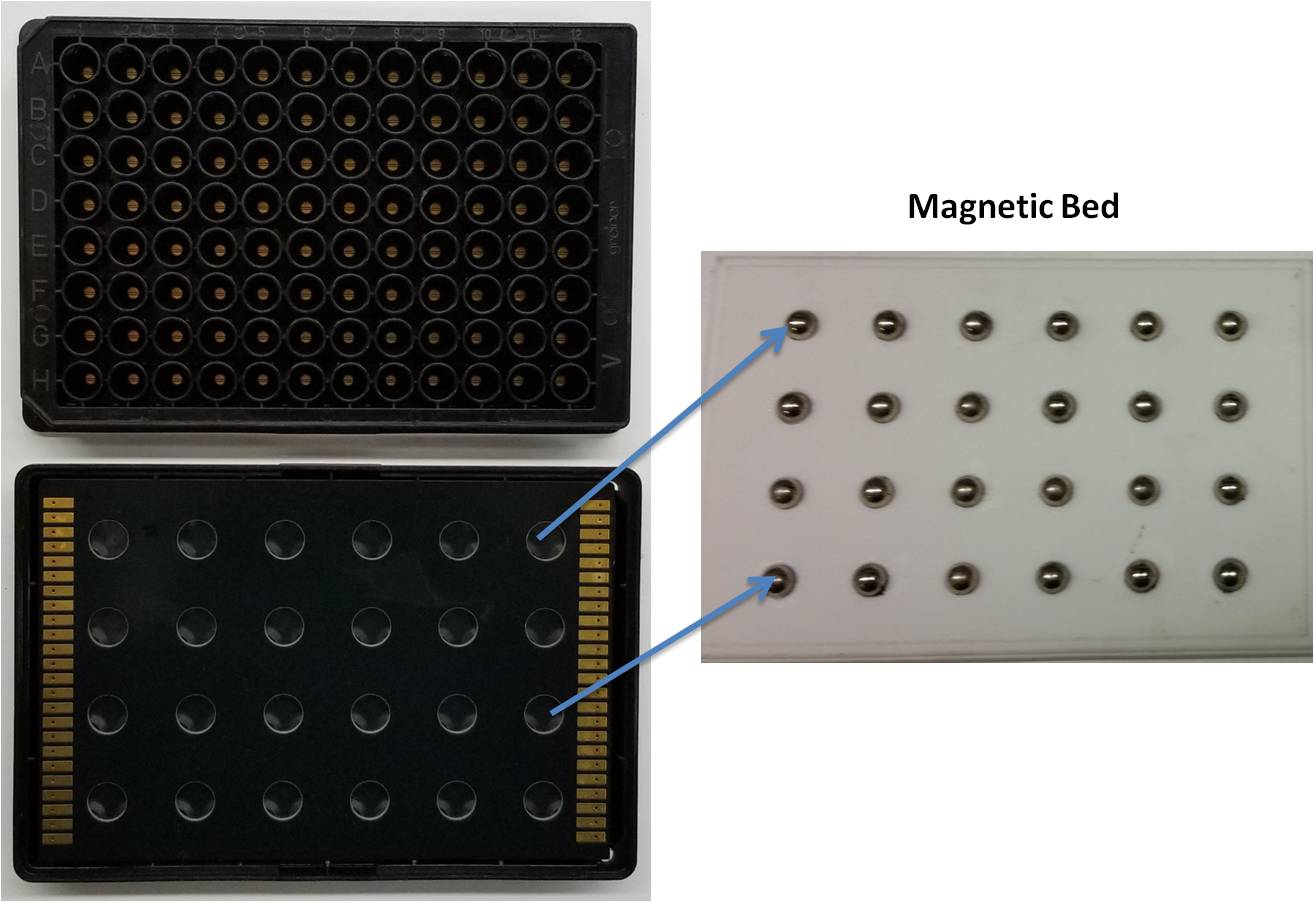


**S1**. a. Examples of the electrode-embedded microwell plates. b. The electrode-embedded black conical plate and the magnetic bed employed for this HbA1c immunoassay.

Three parallel electrodes are arranged along each row of the wells. Parallel orientation of the three electrodes can support the generation of the vortical motion at both sides of the well. Side electrodes have the same widths of about 1000 µm (considering the distance from the center of well to the side walls, the radius of the semicircle formed by the side electodes) and the central electrode is 200 µm wide at the bottom of each well. The distance between the electrodes are both about 200 µm. These parameters are close to the dimensions of the electrode arrangements used in Loire *et al.*’s (39) study where the side electrodes were of 500 µm width, central electrode: 150 µm and gaps between electrodes: 100 µm). In that study, an extensive investigation of the effect of the flow parameters on flow features was conducted.

**Video 1.** Animation of experimental process. It can be reached at “Supporting Information - Video 1”.

**Videos 2.** Motion of micro fluorescent beads in response to driving/varying voltages. It can be reached at “Supporting Information - Videos 2” (0.5V, 1V, 1.5V, 2V, 2.5V, 3V, 3.5V, 4V 1MHz Symmetric mixing videos)
